# Partially Hydrolyzed Guar Gum Increases Ferroportin Expression in the Colon of Anemic Growing Rats

**DOI:** 10.3390/nu9030228

**Published:** 2017-03-03

**Authors:** Luciana Carvalho, Débora Brait, Márcia Vaz, Pablo Lollo, Priscila Morato, Silvia Oesterreich, Jorge Raposo, Karine Freitas

**Affiliations:** 1 School of Health Sciences, Federal University of Grande Dourados, Dourados, Mato Grosso do Sul 79825-070, Brazil; deborarhb@hotmail.com (D.B.); marciasoma@hotmail.com (M.V.); pablo.christiano@gmail.com (P.L.); primorato@gmail.com (P.M.); silviaOesterreich@ufgd.edu.br (S.O.); 2 School of Exact Sciences and Technology, Federal University of Grande Dourados, Dourados, Mato Grosso do Sul 79825-070, Brazil; jorgejunior@ufgd.edu.br; 3 Center of Biological Sciences and Health, Federal University of Mato Grosso do Sul, Campo Grande, Mato Grosso do Sul 79825-070, Brazil; kcfreitas@gmail.com

**Keywords:** dietary fiber, animal model, rats, anemia, iron, growth

## Abstract

Studies have reported a positive effect of prebiotics on the bioavailability of iron. This study evaluated the effect of partially hydrolyzed guar gum (PHGG) on iron absorption mechanisms in anemic rats. Male Wistar rats were fed 75g American Institute of Nutrition Rodent Diets for growth, pregnancy and lactation (AIN93-G) without iron for three weeks in order to induce iron deficiency anemia. Then they were fed a control diet (*n* = 12; without fiber) or a diet with 7.5% of PHGG (*n* = 12), both without iron. Food intake, body growth and the feed efficiency coefficient (FEC) were measured. The animals were euthanized after two weeks of treatment. The weight of the organs, the pH of the cecal content, and the hepatic iron and ferroportin expression in the cecum, duodenum, and liver were assessed. The intake of PHGG reduced food intake without affecting body growth, and there was a difference between the groups regarding the FEC (*p* = 0.026), with the highest value found in the PHGG group. The weight of the cecal content increased (*p* ≤ 0.001) and the pH of the cecal content was significantly lower in the PHGG group. The intake of PHGG significantly increased ferroportin expression in the cecum;however, the difference was not significant in the duodenum and the liver. PHGG seems to have a positive influence on iron absorption through transporter expression, and structural and physiological changes in the colon of anemic growing animals.

## 1. Introduction

Iron deficiency is the most prevalent nutritional deficiency in the world, particularly in developing countries, but it is also common in developed countries [1]. According to the World Health Organization, around two billion people, approximately 30% of the population, are anemic, mainly due to alack of iron, which directly impacts the lives of affected people, especially in the late stages [2,3,4]. Children are among the groups of people vulunerable to iron deficiency anemia due to hematopoiesis, fast growth, and an active metabolic state that requires several micronutrients, especially in the first three years of life [5,6]. Low iron intake, the malabsorption of iron, and monotonous diets with low bioavailability of the mineral, especially in periods of high requirements, are the main causes of anemia [1,6].

The role of some food components, such as prebiotics, has received special attention in the metabolism and absorption of iron [7,8,9]. As described in the literature, prebiotics act on the absorption of minerals by generating short-chain fatty acids forming soluble complexes, increasing the surface absorption, the number of receptors, and the intestinal beneficial bacterial populations, among other mechanisms [10,11].

Partially hydrolyzed guar gum (PHGG) is a soluble fiber, also considered a prebiotic derived from *Cyamopsis tetragonolobus* L., with lower viscosity and the ability to retain a considerable share of the fiber content. It is used on a large scale in enteral feeding, as a dietary fiber supplement, in drinks and foods, and in the food industry in general [12]. However, little is known about the effect of PHGG on the absorption of minerals and its mechanisms, but recent studies have shown the benefits associated with prebiotic intake on mineral absorption [10,12,13], with no damage to the body growth of the animals. The present study aimed to analyze the effect of PHGG intake on the food intake, body growth, intestinal weight, and ferroportin expression in the intestine and liver of anemic growing rats.

## 2. Materials and Methods

Wistar rats (*Rattus norvegicus Wistar*) (*n* = 24) aged 21 days were used at the beginning of the experiment. During the entire study period the rats received ad libitum diet and deionized water by means of the MilliQ Plus system (Millipore Corp., São Paulo, SP, Brazil). They were maintained in individual metabolic cages, made of stainless steel (Beiramar—MA122R^®^, Beiramar, São Paulo, Brazil), in a room with a 12/12 h light/dark cycle, at 23 ± 1 °C. The use of these cages was intended to prevent coprophagy and feed contamination with the iron. This project was entirely approved by the Animal Ethics Committee of the Federal University of Grande Dourados (UFGD) under protocol no 008/2013. Guidelines for the Care and Use of Laboratory Animals in Research were followed.

The animals were fed a diet produced by the researchers based on the diet recommended by the American Institute of Nutrition (AIN93-G). The mineral mix was produced by the Rhoster company without the addition of the 35 mg/kg iron proposed by the AIN93-G diet. Thus, the animals were induced to iron deficiency anemia during the first three weeks of the study (the sixth week of life), and were distributed into two similar groups by weight, body length, hemoglobin rate (Hb), and hematocrit (Ht).

Their weight was measured weekly with a digital balance (Camry EK4052^®^, Zhongshan Camry Eletronic Co., Ltd., Zhongshan, China) with a maximum capacity of 5000 g, and measurement intervals of 1 g, and the length (the body and tail) was measured with a 2 m inelastic measuring tape (Sanny^®^, American Medical of Brasil Ltd., São Paulo, Brazil) with a precision of 0.1 cm, fixed along the bench to facilitate the measurement. Blood collection for hemoglobin and hematocrit was performed on the tail of the animal previously anesthetized with ketamine and xylazine in combination, intraperitoneally (66.6 and 13.3 mg/kg, respectively).

After collection, the samples were taken to the University Hospital of the Federal University of Grande Dourados (HU/UFGD) and analyzed in a Sysmex XT-4000^®^ hematology analyzer (American Medical of Brasil Ltd., São Paulo, Brazil). For confirmation of the induction of anemia, values below 11 g/dL were considered [10]. Therefore, after the induction of anemia, two groups with 12 animals each were formed; the animals were fed the following diets in the subsequent two weeks, as follows: (1) Partially hydrolyzed guar gum group (PHGG): A diet containing 75g of partially hydrolyzed guar gum (100g of Nutrisource Fiber—Nestlé^®^, American Medical of Brasil Ltd., São Paulo, Brazil) and (2) control group: a diet without dietary fiber, which was replaced by 100 g of corn starch, as suggested in the literature [14]. The amount of partially hydrolyzed guar gum was based on a previous study [12], in which there was a recovery of the hemoglobin mass of anemic rats from the addition of this concentration in the diet. It is important to note that both diets had no addition of iron, and the amount of PHGG was subtracted from the total corn starch of the diet, as suggested by the AIN93-G diet.

At first, the food portion prepared was equal to the total amount of food to be ingested by the two groups during the experimental period, consisting of ingredients common to the two groups. Then, the diets were separated, and the individual components of each were added. Most ingredients were weighed in digital balance with a maximum capacity of 10 kg and a precision of 1g (SF-400), and the smallest components were weighed in an electronic analytical balance (BEL Mark M^®^, American Medical of Brasil Ltd., São Paulo, Brazil) with a maximum capacity of 220 g and a precision of 0.1 mg. For confirmation of the iron content, both diets were analyzed by the Laboratory of Spectrometry and Applied Chromatography of the School of Exact Sciences and Technology (FACET) of the Federal University of Grande Dourados (UFGD), measured in triplicate, and their average obtained. The composition of each diet is described in Table 1.

After beginning the administration of the experimental diets, weight, length, and hemoglobin and hematocritvalues were measured on a weekly basis, and food intake was measured daily. In the same period, the feed efficiency coefficient (FEC) was calculated by dividing weight gain (g) by diet consumption (g). After two weeks of the treatment, the animals were euthanized with intraperitoneal injections of ketamine (90 mg/kg) and xylazine (10 mg/kg) in combination. A median incision was made in the abdominal wall and the peritoneum, and then the liver, duodenum, and cecum were removed. In the duodenum, the section removed extended from the pylorus to the ligament of Treitz. The cecum was isolated from the ileocecal valve and the beginning of the proximal colon. All adhesions to the intestines were discarded and the pH of the cecum content was measured. Thus, the animals were euthanized between 6 a.m. and 10 a.m., when intestinal fermentation is more active [10]. The contents of the cecum were placed in a beaker immediately after the body section, and the pH was measured by a benchtop pH meter (Hanna HI 2221-01, Hanna Instruments Brasil, São Paulo, Brazil).

All the organs were rinsed with deionized water to remove excess blood and lumen contents. The organs were placed on filter paper to remove excess water and then weighed on an analytical balance (BEL Mark M^®^). The intestines and the liver sample were stored in a buffer and frozen in an ultrafreezer (−86 °C) until being analyzed by Western blot.

After determining its fresh weight, the remainder of the liver was dried in an oven for 22 h and weighed for three consecutive times or more for a constant dry weight. After drying, an analysis of the hepatic iron content in the samples was performed.

### 2.1. The Determination of Iron in Liver Samples and Diets

Ultrapure water (with a resistivity of 18.2 MΩ cm) obtained from a Millipore Milli-Q Academic deionizer (Millipore^®^, Beadford, MA, USA) was used to prepare all the working solutions.

Iron determination was performed using flame atomic absorption spectrometry AA 240FS (Agilent Technologies^®^, Mulgrave, Victoria, Australia), equipped with a mono element hollow cathode lamp (LCO). Measurements were made at the Fe 248.3 nm line, the lamp current (5 mA), the slit width (0.5 nm), the flame composition (air/acetylene), the acetylene flow (2.0 L/min), the oxidizer flow (13.0 L/min), and the burner height (100 mm). With a flow rate fixed at 5.0 mL/min, the solutions were aspirated by the nebulizer/burner system and the element was atomized under optimal conditions. All measurements were made with five repetitions.

Mono-element calibration analytical solutions were prepared at each daily routine work by appropriate dilution of the stock solution (1000 mg/L, *SpecSol*^®^, SRM-682, Washington, DC, USA) at intervals of 0.5–8.0 mg/L Fe in 1.0% (*m*/*v*) HNO_3_ medium.

For the sample preparation procedure, masses of 1.0000 (±0.0001 g) of the liver and/or diet were weighed in the analytical balance FA2104N (Bioprecisa^®^, Curitiba, PR, Brazil) and transferred to reaction/decomposition tubes. Then, 5.0 mL of HNO_3_ (65% (*v*/*v*), SigmaAldrich^®^, St. Louis, MO, USA) and 3.0 mL of H_2_SO_4_ (98% (*v*/*v*), Vetec^®^, Duque de Caxias, RJ, Brazil) were added. The sample plus the reagent mixture was submitted to slow heating in digestion blocks until it reached 200 °C and it was maintained there for another 150 min. Aliquots of 6.0 and 3.0 mL H_2_O_2_ (30% (*m/v*), Dinâmica^®^, Hexis Científica, Jundiaí, SP, Brazil) were added at 150 and 180 min, respectively. The addition of (H_2_O_2_) helps in the process of decomposition/oxidation present in the samples. Digestion procedures were made in triplicate.

The main parameters of analytical reliability, such as detection and quantification limit, were calculated with the IUPAC (International Union of Pure and Applied Chemistry) method using Microsoft Office^®^ Excel and Microcal^®^ OriginPro (OriginLab©, Northampton, MA, USA) as calculation tools.

Running water was used to clean the materials used in the preparation of the solutions. Later, decontamination was made using immersion nitric acid 10% (*v*/*v*) for 24 h. Then the materials werethoroughly rinsed with deionized water.

### 2.2. Analysis of the Protein Levels by Western Blotting

The tissues (duodenum, cecum, and liver) were homogenized in an extraction buffer (Trizma base 100 mM, pH 7.5; Aprotinin 0.1 mg/mL; phenylmethylsulfonyl fluoride (PMSF) 2 mM; 10 mM sodium Orthovanadate; 100 mM sodium fluoride; 10 mM sodium pyrophosphate, and ethylenediamine-tetraacetic acid (EDTA) 10 M) with a Polytron^®^ model713T homogenizer (Fisatom Equipamentos Científicos, SãoPaulo, SP, Brazil). After homogenization, 10% triton X-100 was added to the samples, and these were kept on ice for 30 min and centrifuged at 15,000 rpm/40 min at 4 °C with a type 70 Ti rotor (Beckman, Fullerton, CA, USA).

The supernatants were collected and the levels of total protein were determined by the Bradford method with a Bio-Rad^®^ reagent (Bio-Rad Laboratório, Hercules, CA, USA). Laemmli buffer was added to the amount of normalized sample by protein concentration (50 μg protein), (0.01% Bromophenol blue, 50 mM sodium phosphate, Glycerol 25%, SDS 1%) containing DTT (200 mM). The microtubes containing the samples were boiled for 5 min and the samples were applied in an 8% polyacrylamide gel (Mini Wide, CA, USA). All gels were marked with ‘rainbow’ mixing patterns from Amersham Bioscience^®^ (Uppsala, Sweden) with the molecular weight compatible with the weight of the proteins to be identified.

An electrotransfer from gel to nitrocellulose membranes was performed for 1.5 h (four membranes) at 15 volts (constant voltage) in a semi-dry transfer tank of Bio-Rad^®^ (Hercules, CA, USA). Then, the membranes were blocked at 4 °C, for 30 min, with 5 mL of blocking buffer, baseline solution (Trizma base 100 mM; EDTA mM; Triton X-100 0.5%; sodium orthovanadate 2 mM), and 3% bovine albumin. The nitrocellulose membranes were incubated overnight at 4°C with a Ferroportin 1 antibody (ferroportin), with a molecular weight of 68 kDa, using Abcam (Cambridge, MA, USA, catalogue number ab58695), in a 1:1000 dilution in a blocking buffer. After 30 min of washing in baseline solution, with stirring every 10 min, the membranes were incubated with the respective peroxidase conjugated secondary antibodies for one hour, in a 1:3000 dilution in a blocking buffer at 22 °C.

Subsequently, the membranes were again washed for 5 min in a baseline solution, which was stirred every 10 min, and were then incubated with an Enhanced Chemiluminescence luminol reagent (ECL) from Amersahm Bioscienses^®^ (Uppsala, Sweden), and exposed to Uvitec Alliance 4.7^®^ gel documentation (Cambridge, UK). The intensity of the bands or spots of interest was identified by the pattern of electrophorectic motility and quantified by optical densitometry using Scion Image Software^®^ (Scion Corporation, Frederick, MD, USA). After the first incubation, the membranes were subjected to stripping with the aim to remove previously bound antibodies and allow re-hybridization with the anti-alpha-tubulin antibody (a protein loading control antibody), with a molecular weight of 56 kDa. Thus, the membranes were incubated with a stripping solution (β-mercaptoethanol 0.2 M; Urea 8 M; albumin 0.05%; Trizma base 1 M), for 30 min, at a temperature of 48 °C, with stirring every five minutes. The membranes were then neutralized with Trizma HCL solution (1 M; pH 7.5) for 30 min at room temperature, also while stirring. Afterwards, the membranes were subjected to blocking, incubation with antialpha-tubulin primary antibody, and then to incubation with a secondary antibody.

The results were expressed as mean ± standard deviation for numerical variables with normal distribution. The student *t*-test was used for comparison between the groups, calculations were performed using Jandel-Sigma Stat software (Systat Software Inc., California, USA), and the level of rejection of the null hypothesis was set at 5% or *p* < 0.05. The Mann-Whitney test was used for numerical variables with non-normal distribution.

## 3. Results

Before the dietary treatment, the average weight of the rats in the PHGG and control groups was as follows: 178.5 ± 26.53 g and 186.5 ± 36.59 g (*p* = 0.560); the body length was 33.42 ± 1.73 cm and 33.47 ± 2.21 cm (*p* = 0.959) and the hemoglobin values were 7.72 ± 1.29 g/dL and 7.69 ± 1.32 g/dL (*p* = 0.963), respectively. Regarding the weight, body length, and hemoglobin, the two groups analyzed in this study were similar before the experimental diets.

### 3.1. Dietary Intake, Body Development, and Feed Efficiency Coefficient (FEC)

Table 2 shows the weekly intake, the total intake in the two-week period, the weekly weight, the body length, and the feed efficiency coefficient (FEC) generated in each period. In the two-week assessment period the PHGG group showed lower values regarding the total intake, which were significantly different from the results of the control group;however, there was no statistically significant difference between both groups in weight and body length. The FEC of the PHGG group was higher than that of the control group at all assessment times, with a statistically significant difference in the first week and the total period.

The total volume of deionized water consumed by the PHGG and control groups during the two-week dietary treatment period was similar, 334.08 ± 75.81 mL and 307.08 ± 49.92 mL (*p* = 0.314). Analysis of the groups at different moments (week 1 and week 2 of the dietary treatment) showed no statistically significant difference between them.

### 3.2. Hemoglobin, Hematocrit, and Hepatic Iron Levels

The weekly hemoglobin (g/dL) and hematocrit (%) values of the two groups after the beginning of the dietary treatment are shown in Table 3. There were no statistically significant differences between the groups, and the results confirmed the induction and maintenance of iron deficiency anemia. The hepatic iron levels, in μg/g, are also presented: there was no statistically significant difference between the two groups.

### 3.3. The Weight of Organs and pH of Cecal Content

The fresh weight of the liver was 11.86 ± 0.85 g in the PHGG group and 11.83 ± 1.45 g in the control group, without statistically significant differences between the groups (*p* = 0.959).

Table 4 shows the weights of the different intestinal sections extracted after euthanasia of the animals. The weight of the cecal wall of the animals in thePHGG group was significantly higher than that of the control group (*p* ≤ 0.001). The pH of the cecal content, which was lower in the group treated with the prebiotic, is also shown (*p* ≤ 0.001).

### 3.4. Ferroportin Analysis

**Figure 1:**
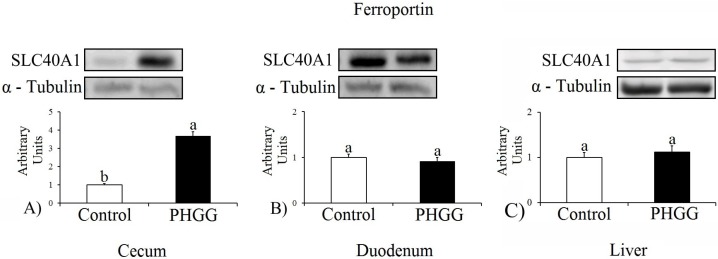
Ferroportin expression. In the cecum, duodenum, and liver of anemic growing rats of the control and the partially hydrolyzed guar gum (PHGG) groups. The protein level was determined by Western blotting and normalized with α-tubulin. The values are means (*n* = 8), with standard deviation represented by vertical bars. Different letters represent statistically significant differences (*p* ≤ 0.001).

Western blotting analysis (Figure 1) identified a significant difference regarding ferroportin in the cecum of the PHGG group compared to the control group (in absolute numbers: 34,902.526 and 9476.214, respectively); however, there was no statistically significant difference between the PHGG and the control groups regarding the duodenum and liver (in absolute numbers, duodenum: 20,956.970 and 22,834.442; liver: 19,660.349 and 19,734.483, respectively).

## 4. Discussion

Dietary fibers are not recommended to children in the first year of life due to their possible impact on growth (early satiety) and mineral absorption [15]. In our study, the rats fed a diet containing 7.5% partially hydrolyzed guar gum showed lower food intake than the control group, although without impact on weight and body length. This finding is similar to the results of another study with PHGG in which the use of fibers reduced, though not significantly, dietary intake without an impact on weight gain in hamsters [16]. In their studies of diets containing 2.5% and 5% PHGG, Yasukawa et al. [17] did not observe reduced dietary intake or loss in weight gain in mice during a four-week monitoring period. The referred studies did not assess body growth. Freitas et al. [12] also did not find significant differences with the use of PHGG on the growth and body weight of growing rats over a three-week period. Other soluble fibers, such as oligofructose and inulin, also did not affect the growth of the animals [7,10,13].

The analysis of hemoglobin and hematocrit during the two weeks of treatment, and of hepatic iron levels at the end of the study, were similar between the groups, without statistically significant differences. It should be stressed that in our study, iron was not added to the diet at any stage of the experiment, which explains the findings. Our primary goal was to observe the effect of the prebiotic on iron inregulatory factors, because anemia is characterized by increased iron regulatory gene expression in the duodenum and the large intestine [18].

The vast bulk of mineral absorption occurs in the small intestine. However, the absorptive capacity of the large intestine has also been reported in the scientific literature. These studies relate to a greater extent to calcium, and to a lesser extent to iron [10,19,20].

It was generally believed that fibers (mostly insoluble) would have a negative effect on mineral absorption because they formed insoluble and stable complexes not absorbed by the gut [15,21,22,23]; however, prebiotics such as inulin and oligofructose are extensively studied in calcium absorption and contradict this principle. The mechanism involved is that the soluble fiber reaches the colon intact and undergoes colonic microbiota fermentation. Such fermentation initiates the intense production of short-chain fatty acids, especially butyrate, consequently reducing the cecal pH and improving the solubility of minerals, facilitating their absorption. In addition, butyrate, the main energy source of colonocytes, induces structural changes stimulating cell proliferation in the entire colon, increasing the absorptive area of the intestinal epithelium [19,24]. Increased mucosal cellularity and the number of intestinal crypts can also contribute to increase the mineral absorptive surface [25].

There are no excretory mechanisms of iron: iron absorption by the small intestine is regulated according to the body’s needs. Thus, low iron intake increases iron regulatory gene expression. The genes involved in iron metabolism, such as Divalent Metal Transporter (DMT1), Cytochrome B Reductase (Dcytb), and ferroportin, are also expressed in the large intestine, but significantly less than in the duodenum [26,27].

Dietary cytosolic iron is exported into the plasma by the basolateral iron exporter ferroportin. The export of iron from enterocytes into the circulation requires the ferroxidase hephaestin, which oxidizes Fe^2+^ to Fe^3+^. In the plasma, Fe^3+^ circulates bound to transferrin (Tf), a glycoprotein that has two binding sites for ferric iron and maintains iron in a soluble form, and is delivered to the bone marrow (for the synthesis of new red blood cells), and the remainder is used for the formation of myoglobin and other proteins (enzyme cofactors) that require additional iron, and also as liver iron reserves [28].

In the present study, the expression of ferroportin, which transports iron into the plasma, was significantly higher in the cecum in the PHGG than in the control group (*p* ≤ 0.001), indicating an increase of approximately 368.3%. There were no statistically significant differences for protein expression in the duodenum and liver. Yasuda et al. [29] explained that the metabolites generated from prebiotics have a greater influence on the expression of genes in the cecum and colon. This study aimed to identify responses to the finding of Freitas et al. [12], and the result is consistent with the findings of these authors who reported that during a three-week experiment, the hemoglobin count was higher in the PHGG group than in the cellulose and control groups (*p* < 0.001).

Sakai et al. [20] investigated the performance of fructo-oligosaccharides (FOS) foriron absorption in the large intestine, monitoring the recovery from anemia in gastrectomized rats with or without cecotomia. The rats were divided into four groups: control (sham operated), gastrectomized (Gx), cecotomized (Cx), and gastrectomized plus cecotomized (GCx). Half of each group was fed a control diet and the other half with a diet containing 7.5% of FOS during 28 days. Only the cecotomized rats showed no decrease in Hb and Ht. The gastrectomized rats showed significantly lower values of Hb and Ht compared to the sham-operated animals, except for the group fed FOS, as this diet prevented anemia in Gx rats. The Hb and Ht of Gx rats fed FOS were statistically higher than in Gx rats fed a control diet (*p* < 0.05);however, in GCx animals, Hb and Ht values were not influenced by the experimental diet. Also, there was an increase in the fraction of soluble iron in the cecal content. Thus, the authors concluded that FOS stimulated iron absorption in the large intestine and that the cecum plays a key role in mineral absorption, especially iron.

Marciano et al. [13], using the same animal model of the present study, assessed the intake of inulin (9.7%) and oligofructose (9.2%) in DMT1, ferroportin, and Dcytb expression. Like PHGG, inulin and oligofructose did not promote a significant increase of these proteins in the duodenum. Inulin increased the DMT1 expression in the cecumby 162%, and the Dcytb expression in the proximal colon by 135% compared to the control group. In the oligofructose group this difference was not significant, with an increase of 59.4% of DMT1 in the cecum and only a 23% increase in Dcytb in the proximal colon compared to the control group. Ferroportin was not significantly increased in the duodenum, cecum or proximal colon in the inulin group and was reduced in the oligofructose group.

Another study [25] involving FOS demonstrated that the prebiotic reduced ferroportin expression in the cecum compared to the other groups. The increase in ferroportin expression in the group fed the PHGG diet may suggest that this fiber is more efficient in increasing the number of iron absorption–related receptors, due to the size of the chain and degree of polymerization, and structural differences of prebiotics may affect iron homeostasis and generate different physiological responses [30]. Thus, further studies are needed to assess the effect of PHGG on other intestinal receptors for comparison purposes.

Our results demonstrated that the fresh weight of liver and duodenum did not differ between the groups, but PHGG and fructans had a similar impact on the significant reduction of pH and onthe significant increase in the cecal area compared to the control group (*p* ≤ 0.001), which would facilitate iron solubilization and absorption. Our study corroborated a study conducted by Freitas et al. [10] using inulin, oligofructose, and synergy (inulin plus oligofructose), in which the liver and duodenum weights did not differ between the groups, but the cecal pH was significantly lower in the groups fed with prebiotics, and the cecal area increased (*p* ≤ 0.001) compared to the group not fed prebiotics.

Although these studies involved animals, they can provide valuable information for humans that would be difficult to obtain in studies with human subjects due to practical and ethical reasons.

In general, studies [10,12,13,30,31] with different types, associations, and concentrations of prebiotics did not observe any negative effect on iron absorption. In contrast with previous evidence, these soluble fibers increased Hb levels or at the very least did not limit iron absorption.

There is little information on the effect of PHGG on mineral absorption. Further studies are needed to clarify the effect of PHGG on other carriers responsible for iron absorption. Although mineral iron absorption by the colon is lower compared to the duodenum, further studies on the use of these fibers by humans may provide greater insight on the physiology of the gastrointestinal tract.

## 5. Conclusions

The data obtained in this study showed that despite the lower food intake by the PHGG group compared to the control group, there was no loss in the weight gain and body growth of the animals. PHGG produced important changes in the cecum of anemic growing animals, as follows: reduced luminal pH, increased cecal wall weight, and greater ferroportin expression. There were no statistically significant differences between the groups regarding the weight of the duodenal wall and liver, the liver iron content and the ferroportin expression in the duodenum and liver during the experiment. Our results demonstrate the benefits of this prebiotic in the expression of proteins regulating iron absorption in the large intestine, facilitating iron uptake, preventing anemia, without impairing body growth.

## Figures and Tables

**Table 1:** Composition of the American Institute of Nutrition Rodent Diets for growth, pregnancy and lactation (AIN93-G) diet indicated for growing rats, modified by replacing 50 g of cellulose and 50 g of starch for 100 g of partially hydrolyzed guar gum (PHGG) or corn starch (control diet) and a free iron mineral mix.

Ingredients	Partially Hydrolyzed Guar Gum (g/kg)	Control (g/kg)
PHGG ^A^	100.000	0.000
Corn starch	479.486	579.486
Casein	200.000	200.000
Saccharose	100.000	100.000
Soybean oil	70.000	70.000
L-cystine	3.000	3.000
Choline bitartrate	2.500	2.500
T-butylhydroquinone	0.014	0.014
Vitamin mix ^B^	10.000	10.000
Free iron mineral mix ^C^	35.000	35.000
Cellulose	0.000	0.000

The composition of macronutrients in 100 g of product: ^A^—Partially hydrolyzed guar gum (100 g of Nutrisource Fiber^®^, Nestlé^®^, Florham Park, New Jersey, USA; 75 g of PHGG): Carbohydrates 0 g; proteins 0 g; total fats 0 g; dietary fiber 75 g; ^B^—(Rhoster^®^, Araçoiaba da Serra, São Paulo, Brazil): Composition in mg: nicotinic acid, 30; pantothenate, 15; pyridoxine, 6; thiamin, 5; riboflavin, 6; folic acid, 2. Composition in μg: Vitamin K, 750; biotin, 200; vitamin B12, 25. Composition in IU: vitamin A, 4000; vitamin D3, 1000; vitamin E, 75; ^C^—(Rhoster^®^): Composition in mg—essential minerals—calcium, 5000; phosphorus, 1561; potassium, 3600; sulphur, 300; sodium, 1019; chlorine, 1571; magnesium, 507; zinc, 30; manganese, 10; copper, 6; iodine, 0.2; molybdenum, 0.15; selenium, 0.15—potentially beneficial minerals—silicon, 5; chrome, 1; fluorine, 1; nickel, 0.5; boron, 0.5; lithium, 0.1; vanadium, 0.1; iron, free.

**Table 2:** The dietary intake, weight, and feed efficiency coefficient (FEC) in the different study periods of the animals in the partially hydrolyzed guar gum (PHGG) and control groups.

	Period	PHGG Group *n* = 12	Control Group *n* = 12	*p*
Dietary Intake (g)	Week 1	127.25 ± 15.05	143.67 ± 10.32	0.002
	Week 2	123.75 ± 16.39	140.75 ± 10.26	0.006
	Total	251.00 ± 28.00	284.42 ± 18.16	0.005
Body Weight (g)	Week 1	222.50 ± 28.40	228.20 ± 35.50	0.670
	Week 2	243.80 ± 27.10	248.80± 34.20	0.695
Body Length (cm)	Week 1	36.07 ± 1.54	36.06 ± 1.83	0.990
	Week 2	37.04 ± 1.55	37.47 ± 1.80	0.542
FEC	Week 1	0.35 ± 0.07	0.29 ± 0.04	0.025
	Week 2	0.17 ± 0.03	0.15 ± 0.03	0.147
	Total	0.26 ± 0.05	0.22 ± 0.04	0.026

*n*: Number of animals. PHGG: Partially Hydrolyzed Guar Gum. FEC: Feed Efficiency Coefficient. The values are means ± standard deviation (Student’s *t*-test).

**Table 3:** Values for hemoglobin, hematocrit, and hepatic iron levels of the animals of the partially hydrolyzed guar gum (PHGG) and control groups in the different study periods.

Variables	Period	PHGG Group *n* = 12	Control Group *n* = 12	*p*
Hemoglobin (g/dL)	Beginning	7.72 ± 1.29	7.69 ± 1.32	0.963 ^1^
	Week 1	8.86 ± 1.50	8,42 ± 1.56	0.823 ^1^
	Week 2	8.65 ± 1.57	8.63 ± 0.99	0.960 ^1^
Hematocrit (%)	Beginning	23.84 ± 5.54	24.42 ± 4.33	0.780 ^1^
	Week 1	25.83 ± 4.75	25.91 ± 4.68	0.969 ^1^
	Week 2	27.70 ± 3.53	27.89 ± 4.04	0.903 ^1^
Hepatic iron (μg/g)	Week 2	115.54 (98.34–128.86)	116.89 (85.38–191.79)	0.751 ^2^

*n*: Number of animals. PHGG: Partially Hydrolyzed Guar Gum. ^1^: Student *t* Test (mean ± standard deviation). ^2^: Mann-Whitney test; median (percentile 25 and 75).

**Table 4:** Weight of the duodenum and cecum and pH of the cecal content in the animals of the PHGG and control groups.

Variables	PHGG Group *n* = 12	Control Group *n* = 12	*p*
Duodenum (g)	0.40 ± 0.09	0.34 ± 0.08	0.139
Cecal wall (g)	1.70 ± 0.25	0.80 ± 0.21	≤0.001
pH of cecal content	5.90 ± 0.57	7.10 ± 0.48	≤0.001

*n*: Number of animals. PHGG: Partially Hydrolyzed Guar Gum. The values are means ± standard deviation (Student *t* test).
